# Supplementary figures and images for: Mitochondrial ROS production by neutrophils is required for host antimicrobial function against Streptococcus pneumoniae and is controlled by A2B adenosine receptor signaling
Source: PLoS Pathog. 2022 Nov 14;18(11):e1010700. doi: 10.1371/journal.ppat.1010700 (PMC9704767; doi:10.1371/journal.ppat.1010700)

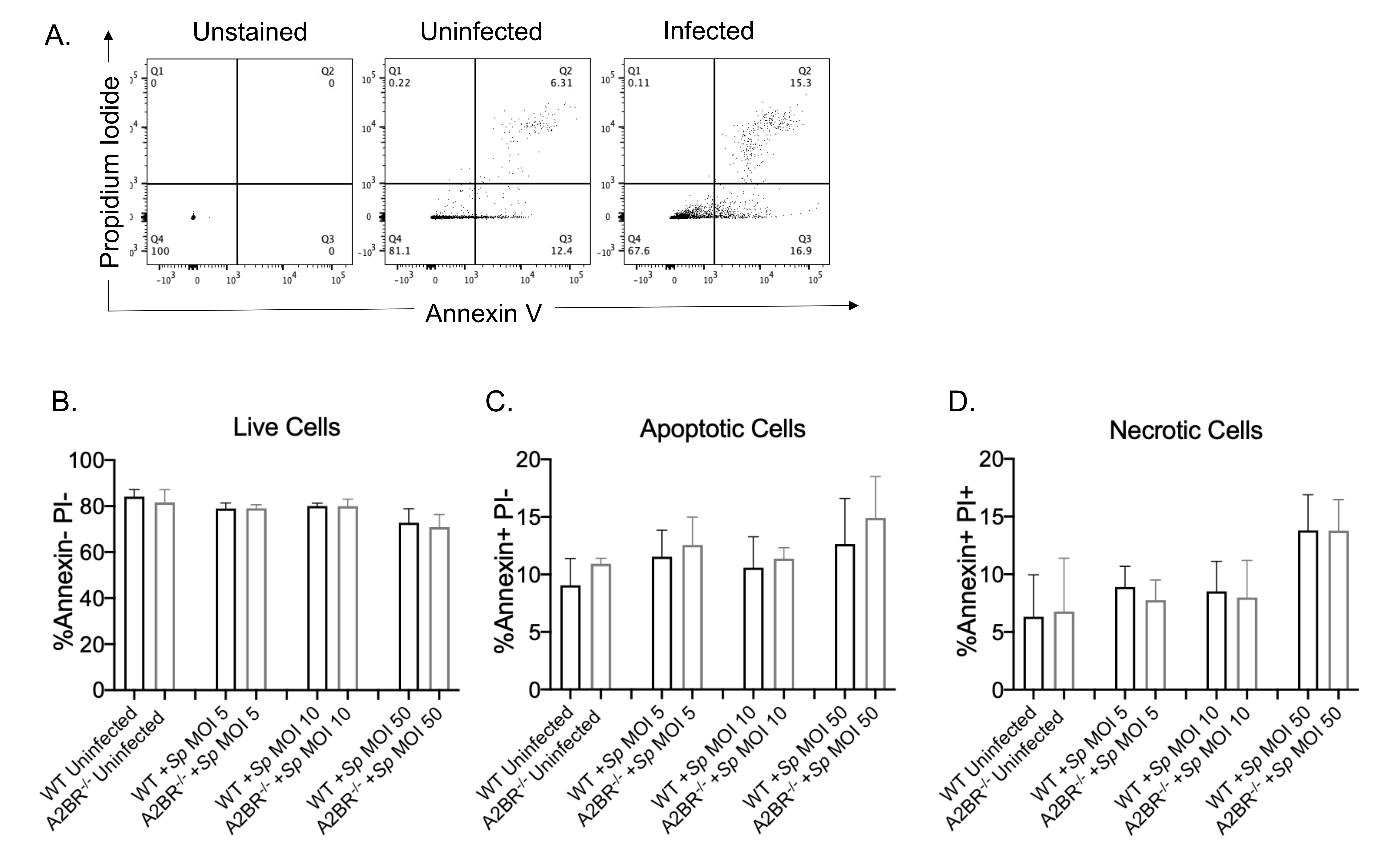

Supplement: S1 Fig — A2BR-/- (light grey) and WT (black) PMNs were mock treated (uninfected) or infected with S. pneumoniae TIGR4 at the indicated MOIs and then stained with Annexin-V and PI. (A) Gating strategy. The % of (B) live (Annexin-, PI-), (C) apoptotic (Annexin+, PI-), or (D) necrotic (Annexin+, PI+) cells was determined by flowcytometry. Bar graphs represent the mean +/-SD. Data are pooled from three separate experiments. (TIF) [file ppat.1010700.s001.tif]
