# Supplementary material for: Mitochondrial ROS production by neutrophils is required for host antimicrobial function against Streptococcus pneumoniae and is controlled by A2B adenosine receptor signaling
Source: PLoS Pathog. 2022 Nov 14;18(11):e1010700. doi: 10.1371/journal.ppat.1010700 (PMC9704767; doi:10.1371/journal.ppat.1010700)
Supplement: Attachment — Submitted filename: Response to Reviwers.docx [file ppat.1010700.s002.docx]

RE: PPATHOGENS-D-22-01132

Response to Reviewers

We would first like to start by thanking the reviewers for their thorough review. Their feedback helped solidify the findings and improved the manuscript. We have addressed all the points in our response here and in the updated manuscript. Based on reviewer feedback, we combined Figures 2-4 examining bacterial factors into new Figure 2, therefore figure numbers have changed. To summarize common points raised by both reviewers:

1. Link between MitROS, A2BR and host resistance *in vivo*: We performed three new studies examining the connection between A2BR, MitROS and host resistance to infection *in vivo.* The data now strongly support the role of A2BR in controlling MitROS production by circulating PMNs *in vivo* and the importance of MitROS in host resistance against systemic *S. pneumoniae* infection.

a. We first examined MitROS production by circulating PMNs following systemic challenge in WT vs A2BR^-/-^ mice over time (0-6 and 18 hpi). We analyzed the number of cells as well as the amount of MitROS produced (MFI). We found that PMNs from A2BR^-/-^ mice produced higher amounts of MitROS compared to WT controls at 6hpi. By 18hpi, the increase in the amount of MitROS and number of PMNs producing it was maintained above background in A2BR^-/-^ but not WT mice (Fig. 9A-C). These findings suggest that A2BR blunts MitROS production by circulating PMNs during systemic disease. These data are now presented in Fig. 9 and in lines 309-326 of the results section.

b. We then tested the role of MitROS production in host resistance against systemic infection in WT mice. We did this using two bacterial strains, *S. pneumoniae* TIGR4 and D39. We found that inhibition of this response increased host susceptibility to systemic infection with both strains, however this was only statistically significant with the D39 strain (Fig. 10 A-E). This could be since WT control mice are extremely susceptible to TIGR4 and thus a further increase in susceptibility is hard to observe even early during infection. Compared to D39, TIGR4 infected mice got sick faster where death observed before 24h (Fig. 10A vs C) and mice had a 10-fold higher bacterial burden even at 6hpi (Fig. 10 B vs D). These data are now presented in Fig. 10 and in lines 328-365 of the results section.

c. We then compared resistance of WT vs A2BR^-/-^ mice following systemic challenge with *S. pneumoniae* TIGR4. We found that A2BR^-/-^ mice displayed significantly enhanced survival and ability to clear the infection compared to WT controls (Fig. 10 C-E). This enhanced protection was lost when mice were treated with a MitROS scavenger (Fig. 10 C-E). These data suggest that A2BR signaling impairs host resistance against systemic *S. pneumoniae* infection and that is in part mediated by controlling MitROS production. These data are now presented in Fig. 10 and in lines 328-365 of the results section.

2. Statistical analysis: We checked the distribution of the data and reran the statistical tests throughout the manuscript. Distribution of the data was tested using the D’Agostino & Pearson and Shapiro-Wilk tests and statistical analysis redone accordingly. This is detailed in the methods section lines 660-667 and in Figure legends. We apologize for the oversight of not doing this in the original submission.

3. Biological replicates: We pooled data to show spread across biological replicates in several figures. All data including bacterial killing by PMNs (New Figs. 4,6) and all animal infection studies (New Figs. 7-10) are now displayed as individual data points where each circle represents one biological replicate (one mouse). To deal with inter-assay variation when measuring MitROS using flow cytometry, as per reviewer suggestion, we presented data comparing mouse strains as fold-change from baseline and pooled the biological replicates in new Figs. 3B (MyD88^-/-^ vs controls) and 5D (A2BR^-/-^ vs WT).

**Part II – Major Issues:**

Reviewer #1: The majority of figures should be updated to show data as dot plots instead of bar graphs, and the statistical tests should be checked (T tests require normal distribution, which do not appear to be appropriate in many cases).

Please refer to points #2 and 3 in the common response above.

Reviewer #2: -Statistics are not shown for Fig 11C to back up conclusion that ‘MitoTEMPO treatment accelerated host death in both strains of mice’, lines 310-311. This is an important point, because Fig 11C provides the only data in vivo demonstrating that the mitochondrial scavenging impacts infection outcomes. While a phenotype is shown with the A2BR-/- mice, the ROS and killing for neutrophils from these mice is only partially dependent on MitROS, so it is important to show that the A2BR and MitROS phenotypes are linked in vivo. It also appears the data in Fig 11C are from one experiment, n=5 mice per group. In this case, the experiment should be repeated to confirm this important finding.

Please refer to point #1 in the common response above.

It is difficult to connect conclusions from the two different infection model systems. For example, what is the interpretation regarding no difference in percent survival for WT vs A2BR-/- mice in the i.p. model (Fig 11), compared to the pulmonary infection where there was a large difference (Fig 9)? To address this concern, it would be helpful to compare blood CFUs for the i.p. model in WT vs A2BR-/- mice, because for the pulmonary model it was shown that A2BR-/- mice have reduced CFUs in the blood but a comparable number of circulating blood PMNs producing MitROS (MitoSOX+). For the i.p. model, there are an increased number of circulating blood PMNs producing MitROS in the A2BR-/- mice but no difference in survival.

We repeated the experiments to get to n=12 mice per group. We now find that A2BR^-/-^ mice displayed significantly enhanced survival and ability to clear the infection compared to WT controls. Please refer to point #1 in the common response above.

S. pneumoniae itself produces ROS including hydrogen peroxide, and live bacteria (or supernatants) are required for MitROS induction (Fig 2). The potential role of bacterial-derived ROS is not addressed, and it is unclear whether this may influence some of the results presented. For example, can MitoSOX detect bacterial ROS? Or, alternatively, is there ROS in the bacterial supernatants and could this contribute to triggering MitROS?

These points have been added to lines 457-459 of the discussion.

**Part III – Minor Issues:**

Reviewer #1: Line 300 – “MitROS production by circulating PMN may be important for controlling systemic spread during S. pneumoniae infection.” MitoROS is similar in both strains in both lung and spleen, and does not correlate with CFU. It is equally likely that MitoROS from PMN has no role in controlling Spn given this data. The authors should adjust this conclusion appropriately. Relatedly, does the level of Mitosox + PMN (in Fig 10C) relate to the CFU of the mice (figure 9B?). i.e. do mice with higher Mitosox levels tend to have more or less CFU?

The wording has been adjusted on new lines 300 of the results section. We now have data directly showing that MitoROS is required for host resistance to systemic pneumococcal infection. Please refer to point #1 in the common response above.

Line 307 / Fig. 11B – “This increase upon infection was significantly different from uninfected controls only in A2BR-/- mice (Fig. 11B).” – This is surprising to me based on the representative plots shown in Fig 11A. The frequency of MitoSOX+ PMN seems to also increase significantly in the WT (3% to 11% is a bigger fold change than seen in figure 10). Please report the frequency in addition the number of cells and perform statistical testing. Also, what were the CFU in the i.p. infected mice (figure 11?)

The % ages show the same trends as the total numbers, therefore we elected to show numbers instead as they are more informative. Although by 18hpi, we see an increase in WT, this was not consistent in all mice. To more thoroughly address this question, we also tracked the response over time and directly assessed the amount (MFI) of MitROS made as well. Please refer to point #1 in the common response above for details.

Line 311 / Fig. 11C – There is no difference in survival between WT and A2BR mice with i.p. infection, so this is a hard experiment to reconcile with the rest of the paper. Looking at the WT alone, with or without MitoTempo, there appears to be no difference in survival, suggesting the mitochondrial ROS is not a main driver of outcome in a normal situation. In contrast, the ABR2 mice with or without MitoTempo looks like it might be significant, but given the spread of the data it is hard to conclude. Do the authors think that theres no role at all, or that there may be a role only in an A2BR-/- mouse (and if so, what would be the reason why?)

We repeated the experiments to get to n=12 mice per group. We now find that A2BR^-/-^ mice displayed significantly enhanced survival and ability to clear the infection compared to WT controls. Please refer to point #1 in the common response above.

There is a difference in survival for i.t. (fig 11 vs fig 9), but not for i.p., - what are the implications for your results? Is it not worth trying the mitoTEMPO in the lung infection model?

We repeated the experiments to get to n=12 mice per group. We now find that A2BR^-/-^ mice displayed significantly enhanced survival and ability to clear the infection compared to WT controls. Please refer to point #1 in the common response above.

Line 312 – is the MitoTempo survival significantly different from the sham treated mice? If not, this data does not support the statement “MitROS are required for host resistance to S. pneumoniae systemic infection.” If they are significant, how do the authors rule out the possibility of a toxic effect of MitoTempo?

We tested this using two bacterial strains, *S. pneumoniae* TIGR4 and D39. We found that inhibition of this response increased host susceptibility to systemic infection but this was more apparent with D39. Please refer to point #1b in the common response above. As a control we included uninfected controls treated with MitoTEMPO and saw no effect on mouse survival. These data are now presented in Fig. 10 and in lines 328-365 of the results section.

Line 317 – “Having established that MitROS production in PMNs are regulated by A2BR signaling” – I think this statement if too bold. The authors show that A2BR-/- leads to increased frequency of MitoSox+ PMN in vitro following stimulation (Fig 7) and that overall, increased levels of ROS (7A) – but I’m not convinced from this manuscript alone that MitoROS is regulated primarily by A2BR.

We changed the wording to “having found” in new lines 368. We also added data showing that A2BR^-/-^ PMNs make more MitROS compared to WT PMNs *in vivo* (New Fig. 9C) and *in vitro* (New Fig. 5C and D). These data are now presented in lines 253-258 and 309-326 of the results section. These findings support the hypothesis that MitoROS is regulated by A2BR.

Line 343 – 344: “we found that recognition of bacterial products is sufficient to trigger this response and that signaling via the extracellular A2B adenosine receptor controls MitROS production”. The clarity of this manuscript would be improved if this part of the discussion briefly summarized the results with a bit more precision, since this sentence builds on results from almost 12 different figures. For example, perhaps something like: we demonstrate that a soluble factor present in culture supernatant is sufficient to induce mitochondrial ROS from PMN… Given that the MyD88-/- cells have reduced, but not abolished MitoSox, and as a specific ligand or signaling pathway was not identified, I think this sentence is a bit strong for the data.

These points have been added to the Discussion lines 443-471. The language has also been adjusted in results section lines 208 and New Fig. 12 legend.

Lines 347-363: Its also possible that in the absence of ROS, MyD88, A2BR etc… is there a differential activation of antimicrobial effectors that lead to differential killing that is independent of ROS? NETosis, degranulation, lysosomal trafficking, autophagy? *note, I see this is addressed later in the discussion (line 373), this should be amended so the writing doesn’t sound as absolute, given the authors are clearly considering the other mechanisms

Lines 475-418 of the Discussion have been amended to clarify this by stating “MitROS can kill bacteria in several ways either directly or by activating other antimicrobial responses.”

Line 375: fmlp should be fMLP

This has been changed throughout the manuscript,

Line 392 onwards: (when discussing the bacterial products and signaling) – is it possible that ATP or adenosine is being released from Spn (for example, following autolysis) that is activating purinergic signaling?

These points have been added to the Discussion lines 457-458.

Line 441 onwards: this appears to be the first time the authors discuss other adenosine receptors. A1 only discussed briefly, while A2A and A3 are not discussed. This should be introduced and explained earlier in the paper (why A2B and not the others) and the discussion would be improved by connecting these new data to their previous work. I am not clear what the authors think the role of each of these receptors are. Further, it may be worth summarizing briefly purinergic signaling in general (can adenosine be released directly, or do the authors assume the pathways is following ATP release and subsequent degradation by ectonucleases?).

The adenosine pathway and other receptors are introduced in the introduction lines 114-128. Under baseline conditions equilibrative transporters keep extracellular adenosine levels low, but adenosine can also be released intracellularly upon cell damage as well. Our prior studies [published in Plos Pathogens in 2015 (PMCID: [PMC4552087](http://www.ncbi.nlm.nih.gov/pmc/articles/pmc4552087/))] found that the exoglycosidase CD73 (as well as CD39) is key for host resistance to pneumococcal infection suggesting adenosine produced in the extracellular environment from breakdown of ATP plays a major role during infection. This point was added to introduction line 123.

METHODS

Line 473 – OD 0.7-0.8 is not mid log (perhaps late log or early stationary). How much autolysis is occurring here?

Mid log has been removed. Based on many growth curves and plating for CFU we have done over the years using the TIGR4 strain, we see exponential increase in viable counts between OD 0.4-1.2. Between OD 1.2-1.5 we see CFUs plateau and after OD 1.5 then crash most likely due to autolysis.

Line 481 – incubation with X% formalin. Was this quenched before adding to cells?

This information has been added to lines 536 of the methods. We used 3% formalin and this was washed prior to addition to cells.

Line 551 – mice were infected with 10-5x104 CFU? Perhaps the authors mean between 1 x104 and 5 x104?

This is clarified on new lines 606 and 623 of the methods. For IT we target 1x10^5^ and IP 1x10^3^. As titers can very within 2-fold we ended up administering 5x10^4^ - 1x10^5^ for IT and 5x10^2^ - 1x10^3^ for IP to the mice across the different experiments performed.

Line 557 (and elsewhere) – ml should be mL (uppercase L, as this is the SI symbol)

This has been changed throughout the manuscript.

Line 563 – dose 1-0.5x104 – what does this mean? Please describe how you determined clincal score

Clinical scoring criteria are now detailed in lines 608-614 of the methods section.

Line 598 – “due to inter-assay variation, representative data are shown”. I understand the challenges with variability but for this reason think the mean from each experiment should be shown. If the raw values are wildly different, perhaps a normalization to media or uninfected control may be useful?

Please refer to point #3 in the common response above.

Line 600 – “log transformed to normalize distribution” – log transformation helps with weighting of the average (prevents skewing) but shouldn’t normalize the distribution of the data. Is this what you mean?

Please refer to point #2 in the common response above.

Fig 6A – the axis break makes this hard to read. I think it would be easier with a single axis going down to -100

This was adjusted.

Fig 7A – this is quite hard to read with so many lines and all black / white. Perhaps moving all the uninfected / control to a second graph would make it less confusing (since there are only really 3 lines that are of interest here? Also, it is not clear what the p<0.0001 means as the line goes across all samples. The legend says this is total ROS. Is this why the MitoTempo doesn’t fully block the signal in the A2BR-/- Spn cells? If so, would like to see this with MitoSox as well.

We added labels to the graph in new Fig. 5A. For MitoTEMPO, yes the response is reduced and not abolished when measuring total ROS as there are likely other ROS sources within the cell. When directly measuring MitROS, addition of MitoTEMPO returns the response back to baseline. This is now added to New Fig. 5B-C and line 257-258 of the results.

Fig 7B and C – Legend: “Representative data shown are from 1 out of 5 separate experiments in which n=3 technical replicates were used per condition.” For 7B and 7C, please include the results from the 5 replicates (ideally with a dot plot and SEM) – was there significant variability in the experiments?

Please refer to point #3 in the common response above.

Figure 8A – please show individual replicates. Significant difference annotation is confusing, does the # p =0.0161 “# indicates significant differences between indicated groups” mean that all three are somehow different, or this is just comparing to WT + VC as well (if so, why this is annotation different from the others?)

Please refer to point #3 in the common response above. For all graphs where we are doing comparison vs baseline and between groups on the same graph, we use * to show difference from the condition tested vs its own baseline and # between treatment groups. This is clarified in all figure legends.

Figure 9A and 9B – T tests assume a normally distributed sample, is that the case for these?

Please refer to point #2 in the common response above.

Figure 9C – Assume this is clinical score of pulmonary pathology? Please clarify in figure legend and include scoring criteria. Representative images would be a nice addition as well.

It is clinical score for disease signs and symptoms. Clinical scoring criteria are now detailed in lines 608-614 of the methods section.

Reviewer #2: -In general, the Figures seem unnecessarily spread out. For example, single panel figures Fig 2, Fig 4, and Fig 5 could be combined, as all address the bacterial components which are responsible for inducing MitROS.

We combined prior Figs 2-4 into a single New Fig, 2.

It is interesting that the MyD88-/- PMNs still had a significant increase in MitROS in response to Sp, though this was not as robust as that induced in WT PMNs (Fig 5). This suggests that MyD88-independent signals also contribute to some degree. This nuance should be incorporated into the Abstract, which states that “MyD88-/- PMNs failed to produce MitROS in response to pneumococcal infection” (line 25), which is not entirely reflective of the data presented in Fig 5.

The wording has been adjusted in the abstract.

Minor comments:

Need to add statistical comparison of (WT + DPI) vs (WT + DPI + MitoTEMPO) in Fig 6A for statement in lines 227-228 stating that there was no additive effect of combining DPI with MitoTEMPO

This was added to New Fig. 4.

Pg 8, line 172-173. This Figure (3A) isn’t testing whether the PMNs are actively infected, more accurately whether they are binding to bacteria

The wording was adjusted on lines 173.

It would be useful to mention in the Discussion that the NLRP3 inflammasome can also induce MitROS, given that the potential bacterial triggers of this response are unresolved.

This has been added to lines 462-465 of the discussion.

Mention of LTA and lipopeptides should be included in the Discussion because they are specified in the Fig 13 model

These were removed from New Figure 12 and replaced with “Bacterial products?”

Show n.s. statistical result for Fig 3A graph

This was added to New Fig. 2B.

Pg 6, line 116 change ‘damage’ to ‘damaged’

This was corrected.

Pg 2, line 23-24 ‘…pneumolysin but presence of live bacteria’ is missing ‘the’

This was corrected.

Pg 13, line 189 change ‘deactivate’ to ‘deactivated’

This was corrected.

Fig 6B shouldn’t have ‘+’ in between 19F and VC and 23F and VC to follow convention
established in other graphs

A + was added to all labels in New Fig. 4 for consistency.

flowcytometry’ needs space between the two words

This has been changed throughout the manuscript.
