# Supplementary material for: Mitochondrial ROS production by neutrophils is required for host antimicrobial function against Streptococcus pneumoniae and is controlled by A2B adenosine receptor signaling
Source: PLoS Pathog. 2022 Nov 14;18(11):e1010700. doi: 10.1371/journal.ppat.1010700 (PMC9704767; doi:10.1371/journal.ppat.1010700)
Supplement: Attachment — Submitted filename: Response to Reviwers.docx [file ppat.1010700.s003.docx]

RE: PPATHOGENS-D-22-01132

Response to Reviewers

We would first like to start by thanking the reviewers for their re-review and comments. Reviewer #2 had no further comments. Here are our responses to the minor comments of reviewer #1.

**Part III – Minor Issues: Editorial and Data Presentation Modifications**
Reviewer #1: extremely minor comments:

Line 241: “Therefore, we investigated the role of the Gs-coupled A2B adenosine receptor in MitROS production by PMNs.” No rationale is provided to support the “therefore.” This sentence can be deleted or augmented with rational.

We edited to clarify the rational on new lines 239-242 as follows: “Signaling via Gs-coupled extracellular adenosine receptors were previously found to inhibit NADPH oxidase activity and ROS production, which led us to investigate the role of the Gs-coupled A2B adenosine receptor in ROS production by the mitochondria in PMNs”

Re Fig 5A (line 248). It is still very hard to discern which line is which in this figure.

We relabeled the figure using the graph symbols and further clarified that all uninfected controls were at blank levels in results lines 245-246.

Line 321-323 / Fig 9C. Authors state there is more MitROS made by the A2BR-/- PMN, but in fig 9C, it doesn’t look striking – can you add the significance comparison here.

We added the p value to the text lines 321. The comparison is also on the graph itself.

Line 336 / Fig 10. Most subfigures in this paper are arranged left to right on the top row, followed by the second row. This one is confusing as it is arranged vertically down a column followed by the second column.

We rearranged Fig. 10 left to right.
